# Role of chemokines in the crosstalk between tumor and tumor-associated macrophages

**DOI:** 10.1007/s10238-022-00888-z

**Published:** 2022-09-29

**Authors:** Rui Qin, Weihong Ren, Guoqi Ya, Bei Wang, Jiao He, Shaoxin Ren, Lu Jiang, Shuo Zhao

**Affiliations:** 1 The First Clinical Medical Institute, Henan University of Chinese Medicine, Zhengzhou, Henan China; 2 Department of Laboratory Medicine, The First Affiliated Hospital of Henan University of Chinese Medicine, Zhengzhou, Henan China

**Keywords:** Tumor-associated macrophages, Chemokine, Tumor, Tumor microenvironment, Targeted therapy, Combination therapy

## Abstract

Tumor microenvironment (TME) consists of a dynamic network of non-tumoral stromal cells, including cancer-associated fibroblasts, endothelial cells, tumor-associated macrophages (TAMs), B and T cells. In the TME, TAMs support tumor initiation, progression, invasion and metastasis by promoting angiogenesis and immunosuppression of the tumor cells. There is close crosstalk between TAMs and tumor cells. Notably, chemokines are a significant messenger mediating the crosstalk between tumor cells and TAMs. TAMs can promote tumor progression via secretion of chemokines. Various chemokines secreted by tumors are involved in the generation and polarization of TAMs, the infiltration of TAMs in tumors, and the development of TAMs' suppressive function. This paper reviews CCL2-CCR2, CCL3/5-CCR5, CCL15-CCR1, CCL18-CCR8, CX3CL1/CCL26-CX3CR1, CXCL8-CXCR1/2, CXCL12-CXCR4/CXCR7 signaling pathways, their role in the recruitment, polarization and exertion of TAMs, and their correlation with tumor development, metastasis and prognosis. Furthermore, we present the current research progress on modulating the effects of TAMs with chemokine antagonists and discuss the prospects and potential challenges of using chemokine antagonists as therapeutic tools for cancer treatment. The TAMs targeting by chemokine receptor antagonists in combination with chemotherapy drugs, immune checkpoint inhibitors or radiotherapy appears to be a promising approach.

## Background

Cancer is one of the major diseases that seriously threaten human health. Statistical analysis of global cancer data in 2020 shows that the incidence and mortality of all cancers are very high [1]. Patients with advanced cancer, in particular, typically have a low five-year survival rate [2]. In the past decades, researchers have tried to develop and explore more effective cancer treatments, but the effects are not significant. During tumor development, the site of tumor growth is called the tumor microenvironment (TME), which plays a crucial mediating role in the interactive relationship between tumor cells and the microenvironment [3]. In TME, tumor-associated macrophages (TAMs) play a vital role [4]. Recruitment and polarization of TAMs are orchestrated by tumor- and host-derived cytokines and chemokines. Among them, chemokines, as key mediators of chemotactic cell recruitment, play a certain regulatory role on TAMs [5]. In this review, we discussed the role of chemokines in the mutual influence between cancer cells and TAMs. Meanwhile, this paper reviews the recent progress in the treatment of tumors with chemokine antagonists, showing that drugs targeting chemokines can be useful in modulating the effects of TAMs.

## Overview of chemokines 

Chemokines are low molecular weight chemotactic proteins (8–10 kDa) that regulate leukocytes trafficking to the inflammatory site [6]. The chemokine receptors, a member of the G protein-coupled receptor family, bind chemokines and transmit signals via GTP-binding proteins[7]. Chemokines bind to G protein-coupled receptors to regulate cell cellular adhesion, proliferation, migration, as well as inflammatory mediators expression.

In TME, chemokines and their receptors can be expressed in a variety of cells, including tumor cells, endothelial cells and immune cells [6]. On the one hand, chemokines often lead to the recruitment of pro-tumorigenic immune cells, such as myeloid-derived suppressor cells (MDSCs), TAMs, tumor-associated neutrophils (TAN) and regulatory T cells (Tregs). These recruited leukocytes induce tumor immune escape, stimulate tumor growth as well as enhance the proliferation and migration of tumor cells [8]. On the other hand, chemokines inhibit tumor invasion, growth and metastasis by mediating antitumor immune responses, such as recruiting CD4 + T cells, CD8 + T cells and natural killer cells (NK cells) [8]. In summary, chemokines further influence tumor progression and therapy by mediating tumor immunity.

## Classification of TAMs and their biological functions

**Fig. 1:**
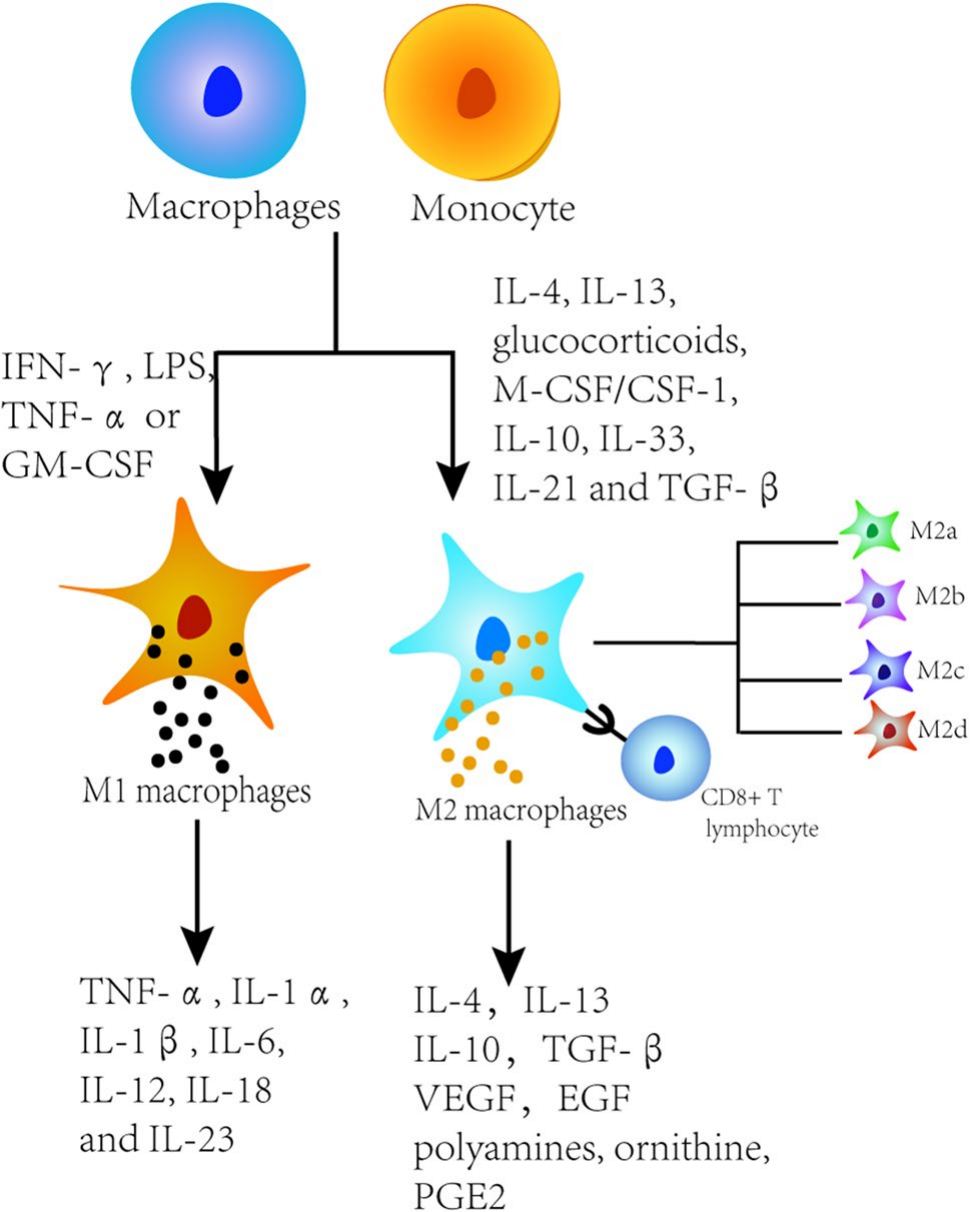
Cellular origins and functions of TAMs

Macrophages existing in the TME are called TAMs. Resident tissue-specific macrophages and newly recruited monocytes are recruited into the TME and differentiate into TAMs under the influence of growth factors and chemokines produced by tumor cells and tumor stromal cells [9]. TAMs are known to be polarized into two phenotypes, M1 (classically activated) and M2 (alternatively activated) TAMs, which play different roles in TME (Fig. 1) [10].

Resident tissue-specific macrophages and newly recruited monocytes differentiated into M1 and M2 macrophage phenotypes. Proinflammatory M1 can be activated by IFN-γ, lipopolysaccharide (LPS), tumor necrosis factor-α (TNF-α) or GM-CSF, which subsequently activate Toll-like receptor signaling pathways. Anti-inflammatory M2 macrophages can be induced by cytokines such as IL-4, IL-13, glucocorticoid, M-CSF/CSF1, IL-10, IL-33, IL-21 and transforming growth factor-β (TGF-β). M2 macrophages develop further and can be subdivided into M2a, M2b, M2c and M2d subgroups. Proinflammatory M1 promotes type 1 T helper (Th1) antitumor immune response by producing cytokines such as TNF-α, IL-1α, IL-1β, IL-6, IL-12, IL-18 and IL-23. M2 macrophages increase the production of polyamines and ornithine via the arginase pathway, secrete high levels of IL-10, PGE2 and TGF-β, but low levels of IL-12, and participate in Th2 immune response, including humoral immunity, anti-inflammatory and wound healing. In solid tumors, M2 macrophages promote tumor progression and invasion by inducing angiogenesis and suppressing the host immune response.

Macrophages activated by the classical pathway are of the M1 phenotype. M1 macrophages secret cytokines and chemokines to regulate acute inflammation, which play a major role in inhibiting tumor growth [11]. Macrophages produced by alternating activation are of the M2 phenotype. M2 macrophages secrete IL-4/10 and other anti-inflammatory factors, regulate the secretion of vascular endothelial growth factor (VEGF), promote angiogenesis, lymphangiogenesis, tissue reconstruction and damage repair, as well as inhibit immune responses via Th2 cell responses, contributing to tumorigenesis and metastasis [12]. M2 macrophages develop further and can be subdivided into M2a, M2b, M2c and M2d subgroups. Their specific characterizations have been reviewed by Abbas Shapouri Moghaddam et al. [13]. In TME, tumor produces a large number of cytokines and growth factors such as IL-4, granulocyte–macrophage colony-stimulating factor (GM-CSF). The production of these cytokines and growth factors can induce the transformation of most TAMs from M1 macrophages to M2 macrophages in TME [14]. Recent studies have found that TAMs have a subgroup of monocyte macrophages that express Tie2 called TEMs (Tie2-expressing monocyte/macrophages). TEMs highly express the pro-angiogenic factor such as matrix metalloprotein-9 (MMP-9), VEGF and M2 markers such as cyclooxygenase-2 (COX-2), CD206. Meanwhile, they are more prone to M2 polarization than TAMs and have a greater capacity to promote vascular remodeling [15].

In conclusion, TAMs are more readily polarized to M2 macrophages based on its role in the TME. As an important component of the tumor stroma, TAMs accumulate around blood vessels to induce angiogenesis and promote tumor invasion. In addition, they modulate the immune system and promote the development of milieu for immunosuppression that counteracts immune responses.

## CC chemokines

There are 28 types of chemokines (CCL1-CCL28) belonging to the CC chemokine subfamily, among which CCL2, CCL3, CCL5, CCL15, CCL18 and CCL26 exert some regulatory effects on pathological processes such as TAMs infiltration and polarization in cancer [16, 17].

### CCL2-CCR2

CCL2, also known as MCP-1, is primarily secreted by macrophages, monocytes, dendritic cells (DCs). The binding of CCL2 and its receptor CCR2 plays a central role in macrophage-related functions by mediating cancer-related inflammation, regulating the proportion of M1 and M2 macrophages, promoting the recruitment of TAMs and providing anti-apoptotic or angiogenic signals [18, 19].

In several tumor types including retinoblastoma, activation of the CCL2-CCR2 axis promotes the recruitment of TAMs and MDSCs into the TME [20]. Meanwhile, CCL2 is an important regulator of the recruitment of CCR2 + inflammatory monocytes (IM) from the bone marrow into the peripheral blood, where they eventually polarize into immunosuppressive TAMs [21]. More importantly, in a mouse model of esophageal squamous cell carcinoma (ESCC), M2 polarization increased PD-L2 expression in TAMs, leading to immune evasion and tumor promotion via the programmed cell death protein 1(PD-1) signaling pathway. Blockade of the CCL2-CCR2 axis strongly reduces the incidence of tumor by inhibiting TAMs recruitment and enhancing the anti-tumor efficacy of CD8 + T cells in the TME [22, 23]. Therefore, the CCL2-CCR2 axis is promising as an anticancer therapeutic target because of its key role in TAMs recruitment and tumor progression.

### CCL3-CCR5/CCR1 

CCL3, a macrophage inflammatory protein 1α, is secreted by monocytes and macrophages [24, 25]. The CCL3-CCR5/CCR1 signaling pathway plays an important role in the process by which TAMs influence tumor development.

In pediatric high-grade gliomas (pHHGs), CCL3 was found to be a key mediator of TAMs infiltration. After CCL3 gene was knocked out in a mouse model of pHHGs, TAMs infiltration was inhibited and the mice had prolonged survival [26]. In addition, CCL3 binding to the CCR5 receptor enhances the ability of TAMs to promote tumor cell invasion and metastasis. When the CCL3-CCR5 signaling pathway is blocked, the ability of TAMs is inhibited [27]. The interaction of CCL3-CCR1 is also a tumor-promoting signal. It was found that CCL3-CCR1 signaling pathway can recruit inflammatory monocytes into the TME and differentiate into M2 macrophages [28]. Moreover, M2 macrophages regulate TME by the secretion of CCL3 and so on [29]. Treatment of cells with antagonists of the CCL3 receptors CCR1 and CCR5 or with antibodies against CCL3 significantly inhibited the macrophage migration [30]. Therefore, CCL3 may be a potential target for anticancer therapy alone or in combination. However, CCL3, as a marker of M1 macrophages, promotes M1 polarization of TAMs [31] and CCL3-CCR1 signaling in turn promotes M2 macrophage generation [28]. Therefore, whether CCL3 chemokine signaling promotes M1 polarization or M2 polarization in tumors needs to be judged according to tumor types and the characteristics of macrophages.

### CCL5-CCR5 

CCL5 is mainly expressed in T lymphocytes, macrophages and some types of tumor cells [32]. It can recruit a variety of leukocytes such as T lymphocytes, monocytes/macrophages, to the sites of injury and infection [33]. The chemokine CCL5 and its receptor CCR5 are pivotal players in the development of tumor affected by TAMs.

It has been found that malignant lobular tumors recruit and repolarize TAMs to drive the malignant progression via the CCL5-CCR5-driven signaling cascade [34]. Yuan et al. found that CCL5-CCR5 signaling pathway induced the polarization of TAMs into M2 phenotype, resulting in reduced sensitivity of liver cancer cells to X-rays [35]. In addition, as a TAM mediator, CCL5 promotes tumor proliferation, invasion and metastasis [36]. And TAMs enrichment has been found in chemo-resistant prostatic tumor tissues. Those TAMs are confirmed to promote chemo-resistance and distant metastasis in prostatic cancer by secreting CCL5 [37]. As reported for gastric cancer, TAMs are capable of promoting cancer progression by activating CCL5/CCR5/STAT3 signaling [38]. In breast cancer-related studies, it has been found that the CCL5-CCR5 axis plays a key role in the metabolic communication between cancer cells and macrophages [39]. Thus, blocking CCL5-CCR5 interaction (using CCR5 inhibitor maraviroc, blocking CCL5 expression, using anti-CCR5 antibody or CCL5-neutralizing antibody) can reduce the number and immunosuppressive potential of tumor invasive TAMs, inhibit tumor metastasis and improve the survival rate of tumor-bearing mice [34, 40–42]. So, targeting the CCL5-CCR5 axis may be a new strategy for antitumor therapy.

### CCL18-CCR8 

CCL18 is a marker of M2 macrophages. TAMs promote immunosuppression and immune escape of tumors by producing CCL18. Due to its immunosuppressive characteristics, CCL18 is not only a marker, but also related to the characteristics of M2 macrophages [43].

In gallbladder cancer, the CCL18 chemokine secreted from M2 macrophages activates PI3K/AKT signaling and leads to cell migration, invasion and epithelial–mesenchymal transition (EMT). Blocking the function of CCL18 with a neutralizing antibody reversed these effects [44]. It has also found that M2 macrophages induced the EMT of ovarian cancer cells by releasing CCL18 in the globules [45]. In colorectal cancer [46], osteosarcoma [47], head and neck squamous cell carcinoma [48, 49], breast cancer [50–52], pancreatic ductal adenocarcinoma[53], lung cancer[54], etc., M2 macrophages all promote tumor invasion and metastasis by secreting CCL18. In addition, some studies have found that CCL18 antagonist can block tumor metastasis [55]. Meanwhile, it was found in breast cancer that M2 macrophages promote tumor angiogenesis by secreting CCL18 [56]. And sequencing showed that CCL18 + TAMs play an immunosuppressive role by inhibiting the production of inflammatory factors in non-small cell lung cancer [57]. In conclusion, M2 macrophages secrete CCL18 to promote tumor invasion and metastasis, tumor angiogenesis and immunosuppressive function, so as to promote tumor progression.

### Other CC chemokines 

CCL15, also known as leukotactin-1 (LKN-1), is produced by macrophages and neutrophils and acts by binding to CCR1 [58, 59]. Elevated levels of CCL15 in serum and tumor tissue resulted in the accumulation of TAMs in tumor tissues. It was found that CCL15 produced by follicular thyroid cancer cells is responsible for the recruitment of TAMs, which could be inhibited by treatment with a CCL15 blocking antibody [60]. Moreover, M2-phenotype TAMs promote tumor cell resistance through paracrine CCL15 [61]. In short, CCL15 is able to promote the accumulation of TAMs in the TME and increase the drug resistance of tumor cells, so CCL15 may be used as a new target for anti-tumor therapy.

CCL26, also known as eosinophil chemokine-3, is mainly expressed by macrophages and epithelial cells, chemotactic on eosinophils, monocytes and T cells [62–65]. It exerts its effect by binding to CX3CR1. Regenerated liver phosphatase 3 (PRL-3) has been found to promote the invasion and metastasis of colorectal cancer by upregulating CCL26 to induce TAMs infiltration [66]. However, the regulatory effect of CCL26-CX3CR1 chemokine signal transduction on TAMs polarization needs further study.

## CXC chemokines

There are 17 chemokines (CXCL1 ~ CXCL17) in the cxc-type chemokine subfamily, of which CXCL8 and CXCL12 can regulate TAMs in tumors [16, 17].

### CXCL8 (IL-8)-CXCR1/CXCR2 

CXCL8, also known as IL-8, is a cytokine critical for immune cell recruitment and activation, attracting neutrophils, macrophages, DCs and endothelial cells by binding to its receptors CXCR1 and CXCR2 [67].

CXCL8-CXCR1/2 axis appears critical for TAMs mobilization and recruitment in the TME and is highly expressed in the tumor [68, 69]. For instance, circulating hypoxia activated hypoxia-inducible factor 1(HIF-1) and NF-κB in tumor cells, which resulted in increased production of VEGF-A, CCL2/MCP-1, CXCL1, CXCL8/IL-8 and prostaglandin E2(PGE2). Subsequently, these factors recruit neutrophils and monocytes into the tumor niche, where they are converted into TANs and TAMs [70]. In addition, studies have shown that the infiltration of TAMs in the TME leads to the increase of CXCL8 and enhances tumor invasion and angiogenesis [71]. CXCL8 can also decrease CD8+ T cell infiltration and increase PD-L1 expression in macrophages, thereby inhibiting CD8+ T cell activity and playing an important role in gastric cancer immunosuppression [72]. It has been demonstrated that CXCL8 secreted by TAMs enhanced the migration, invasion and EMT capabilities of breast cancer cells. These abilities were significantly inhibited when treated with an antagonist of CXCR2 (Danirixin) [73]. Meanwhile, IL-8 exhibits a promoting effect on the differentiation of monocytes into CD206+ TAMs, and the concentration of IL-8 is increased in tumors [74]. Anti-IL-8 monoclonal neutralizing antibody can interfere with the transition of peripheral blood monocytes to TAMs [75]. Currently, it has been found that interferon-γ (INF-γ) inhibits TAMs tumor trafficking, which is mediated by CXCL8-CXCR2 axis [76]. Therefore, researchers can develop targeted drugs for tumor therapy based on the regulation of TAMs by CXCL8.

### CXCL12-CXCR4/CXCR7

CXCL12, also known as stromal-derived factor (SDF-1), can bind to CXCR4 and CXCR7 [77]. It induces monocytes migration, and monocytes then differentiate into macrophages that shape the immunosuppressive microenvironment and support tumor cell proliferation and angiogenesis [78, 79].

Tumors induce the migration and recruitment of TAMs through the CXCL12-CXCR4 signaling pathway [80]. CXCR4-CXCL12 is also an important signal transduction axis involved in TAMs polarization [81, 82]. Polarization of M2 phenotype to M1 phenotype is promoted when CXCL12 and CXCR4 expression is downregulated in tumor cells [83]. TAMs also enhance the survivability of cancer cells through CXCL12-CXCR4 signaling [84]. Meanwhile, CXCL12 and its receptor may contribute to TAMs-mediated CD8^+^ T cell suppression [85]. In addition, tumor cell infiltration requires a unidirectional transition from migratory to perivascular macrophages, which is regulated by CXCL12 and CXCR4 [86]. In a murine model of ovarian cancer, dual blockade of the CXCL12-CXCR4 and PD-1-PD-L1 signaling cascades effectively promotes polarization of M2 to M1 macrophages in tumors [87]. The downregulation of CXCL12 in stromal cells by pro-epigallocatechin-3-gallate (EGCG) treatment impeded macrophages migration and differentiation, thereby inhibiting the infiltration of VEGFA-expressing TAMs [88]. In the treatment of glioblastoma, a novel SDF-1α inhibitor, olaptesed pegol (OLA-PEG, NOX-A12), was developed to reverse the recruitment of TAMs in the TME [89]. Therefore, targeting the CXCL12-CXCR4 axis to modulate TAMs may be a potential target for anticancer therapy alone or in combination.

## CX3C and XC chemokines 

CX3CL1 is the only chemokine of the CX3C chemokine subfamily [90–92]. It regulates the infiltration and polarization of TAMs in tumors [93]. The CX3CL1-CX3CR1 axis promotes tumor progression by recruiting M2 phenotype as well as regulating the role of TAMs in the development of skin cancer and liver cancer. When neutralizing antibodies against CX3CL1 were used, the migration and invasion abilities of cancer cells were weakened [94, 95]. In breast cancer and testicular germinal tumor, CX3CL1 mediates the infiltration of TAMs, resulting in poor tumor prognosis, but the specific types of TAMs are unclear and further research is needed [96, 97].

When monocytes differentiate into TAMs, CX3CR1 is activated, and activation of this receptor inhibits the apoptosis of TAMs, leading to an increased number of TAMs [98]. Moreover, CX3CL1 may increase platelet-derived factor 4 (PF-4)/CXCL4 production in macrophages, thereby enhancing VEGF-mediated angiogenesis [99]. However, the direct effects of CX3CL1 on the pro-angiogenic properties of macrophages still require thorough investigation. Because CX3CL1 has been shown to decrease VEGF-A production by the GM-CSF-stimulated macrophages, at the same time inhibits angiogenesis [100].

The XC-type chemokine subfamily includes two chemokines, XCL1 and XCL2. XCL1 is a specifical and critical player in the tissue-specific recruitment of T lymphocytes [101]. XCL2 specifically induces TAMs to differentiate toward M1 phenotype [102]. At present, the relationship between XCL1/XCL2 and TAMs in tumor needs to be further studied.

## Chemokine antagonists against TAMs survival 

In recent years, scholars have found that chemokines and their receptors can influence the body's physiology and pathology, and play an important role in tumors and other diseases. Different studies have identified that a variety of chemokines and their receptors play a certain role in regulating the recruitment, infiltration and polarization of TAMs in the TME. In the meanwhile, TAMs mediate tumor development and metastasis via chemokine signaling pathways. These chemokine ligands and receptors are potential therapeutic targets to prevent tumor cells spreading. Bl5923, an antagonist of CCR1, has been found to suppress liver metastasis of colon cancer [103]. The antagonist of CCR1, CCX721, also reduces tumor burden and osteolysis in a mouse model of myeloma bone disease [104]. Treatment of immunodeficient mice with a CCR2 antagonist (RS504393) greatly suppressed the infiltration of TAMs [105]. Another CCR2 antagonist (RS102896) inhibits estrogen-induced liver metastasis of michigan cancer foundation-7 (MCF-7) human breast cancer cells [106]. The CCR5 antagonism of Maraviroc decreases metastases in gastric [107] and breast cancer [108]. Although clinical trials of chemokine receptor antagonists in tumor are still limited, some effective drugs have been developed. For example, pF-04136309 and the CCR2 antagonist CCX872 mentioned above have made some progresses in clinical trials. A CCR5 antagonist (Maraviroc) in combination with chemotherapy has been demonstrated to prolong overall survival in a small-scale phase I clinical trial (ClinicalTrials.gov ID: NCT01736813) in metastatic colorectal cancer patients [109].

Despite these encouraging results, treatment with a single chemokine antagonist is insufficient to inhibit the growth of metastatic tumors. A tumor expresses a variety of chemokines and their receptors, and only antagonists or antibodies acting on many chemokine receptors can significantly inhibit tumor development and metastasis. A bispecific single-domain antibody that specifically binds to CCL2 and CCL5 was discovered in therapeutic studies of liver malignancies. It induces the polarization of TAMs toward the antitumor M1 phenotype and reduces immunosuppression in the TME. When this antibody was combined with a PD-1 ligand inhibitor, the survival time of a mouse model of liver malignancy was significantly increased [110]. In the treatment of pancreatic ductal adenocarcinoma (PDAC), when a dual antagonist of CCR2 and CCR5 (BMS-687681) was used in combination with αPD-1 or radiotherapy (RT), the infiltration of M2 macrophages, MDSCs and regulatory T cells was inhibited. The combination therapy led to better survival and tumor control [111]. A mouse model where MC38 colon cancer cells were grown intramuscularly has demonstrated that cenicriviroc (dual CCR2 and CCR5 antagonist) treatment inhibited TAMs accumulation. Further, targeting this cell population using a dual antagonist of CCL2 and CCL5 improves the efficacy of RT overall [112]. All of the above suggest that dual-chemokine receptor antagonists or dual-chemokine antibodies are attractive drugs for cancer therapy. However, the clinical use of dual antagonists in tumors requires further identification of chemokine signal combinations that function under different conditions (e.g., tumor origin, metastatic site and stage of progression). In addition to selecting appropriate targets, it is also important to determine the functional dose of antagonists sufficient to provide continuous receptor coverage in vivo.

However, using chemokine antagonists to block the accumulation of TAMs is unlikely to induce tumor cell death directly. Therefore, macrophage-targeted therapy should be combined with others such as chemotherapy or immunotherapy, which may be a better way to directly kill cancer cells. It is found that targeting TAMs by a CCR2 inhibitor (pf-04136309) in combination with the standard chemotherapy regimen FOLFIRINOX reduced the infiltration of TAMs and Tregs as well as increased the number of CD4 + and CD8 + effector cells [21]. It has also been found that CCR2 antagonist (CCX872), when combined with FOLFIRINOX, increased overall survival and decreased peripheral blood monocyte counts in patients with locally advanced/metastatic PDAC [113]. CCR5 antagonists (maraviroc, leronlimab) and CXCR4 antagonists (balixafortide, burixafor, GMI-1359, motixafortide) have also been shown to synergistically inhibit tumor development in combination with chemotherapy [114–119]. These results suggest that elimination of macrophages through chemokine receptor antagonists in combination with direct cancer cell killing by chemotherapy is an effective therapeutic strategy to prevent malignant tumor development. However, macrophages blockade may not always enhance chemotherapy efficacy. Studies have found that the use of CCR2 antagonists may result in a reduced ability to block the accumulation of TAMs, without enhancing the efficacy of chemotherapy [120]. These results suggest that a certain therapeutic approach affects the characteristics of macrophages in tumors, and therefore the use of chemokine receptor antagonists in other therapeutic modalities should be carefully evaluated.

Anti-PD-1/PD-L1, as classic immune checkpoint inhibitors, mainly exerts their effects by facilitating the activation of tumor-specific cytotoxic T cells. TAMs have been reported to inhibit the function of CD8^+^T and NK cells in vitro. Therefore, targeting TAMs is of great significance to improve the efficacy of anti-PD-1/PD-L1 immunotherapy. Currently, intervene with chemokines secreted by TAMs is a well therapy. Selective chemokine/chemokine receptor inhibitors have been developed to enhance the responsiveness of immune checkpoint inhibitors by modulate TAMs. For example, CCR2 antagonists (BMS-813160), CCR5 antagonists (maraviroc, vicriviroc) and CXCR4 antagonists (motixafortide, mavorixafor) have been found to enhance anti-PD-1/PD-L1 efficacy [114, 115, 119, 121, 122]. These results suggest that elimination of macrophages can effectively improve the efficacy of anti-PD-1/PD-L1 immunotherapy. Thus, TAMs blockade by chemokine receptor antagonists in combination with anti-PD-1/PD-L1 immunotherapy may be a promising therapy to prevent the progression of malignant tumors. But chemokines also play a recruiting role for cytotoxic lymphocytes, so they should be chosen with caution when selecting chemokine receptors. The above combination therapies are summarized in Table 1.

**Table 1:** Combination therapies of the TAMs targeting by chemokine receptor antagonists with other therapies

Targeting pathways and mechanisms	Active drugs	Combination therapy	References
CCL5/CCR5	Maraviroc	PD-1, Chemotherapy	[114]
CCL5/CCR5	Leronlimab	Chemotherapy	[115]
CCL5/CCR5	Vicriviroc	PD-1	[115]
CXCL12/CXCR4	Balixafortide	Chemotherapy	[116]
CXCL12/CXCR4	Burixafor	Chemotherapy	[117]
CXCL12/CXCR4	GMI-1359	Chemotherapy	[118]
CXCL12/CXCR4	Motixafortide	Chemotherapy, PD-1, PD-L1	[119]
CCL2/CCR2、CCR5	BMS-813160	PD-1, chemotherapy, vaccination	[121]
CXCL12/CXCR4	Mavorixafor	PD-1	[122]
CCL2/CCR2	CCX-872	Chemotherapy, radiotherapy	[123]

## Concluding remarks

**Fig. 2:**
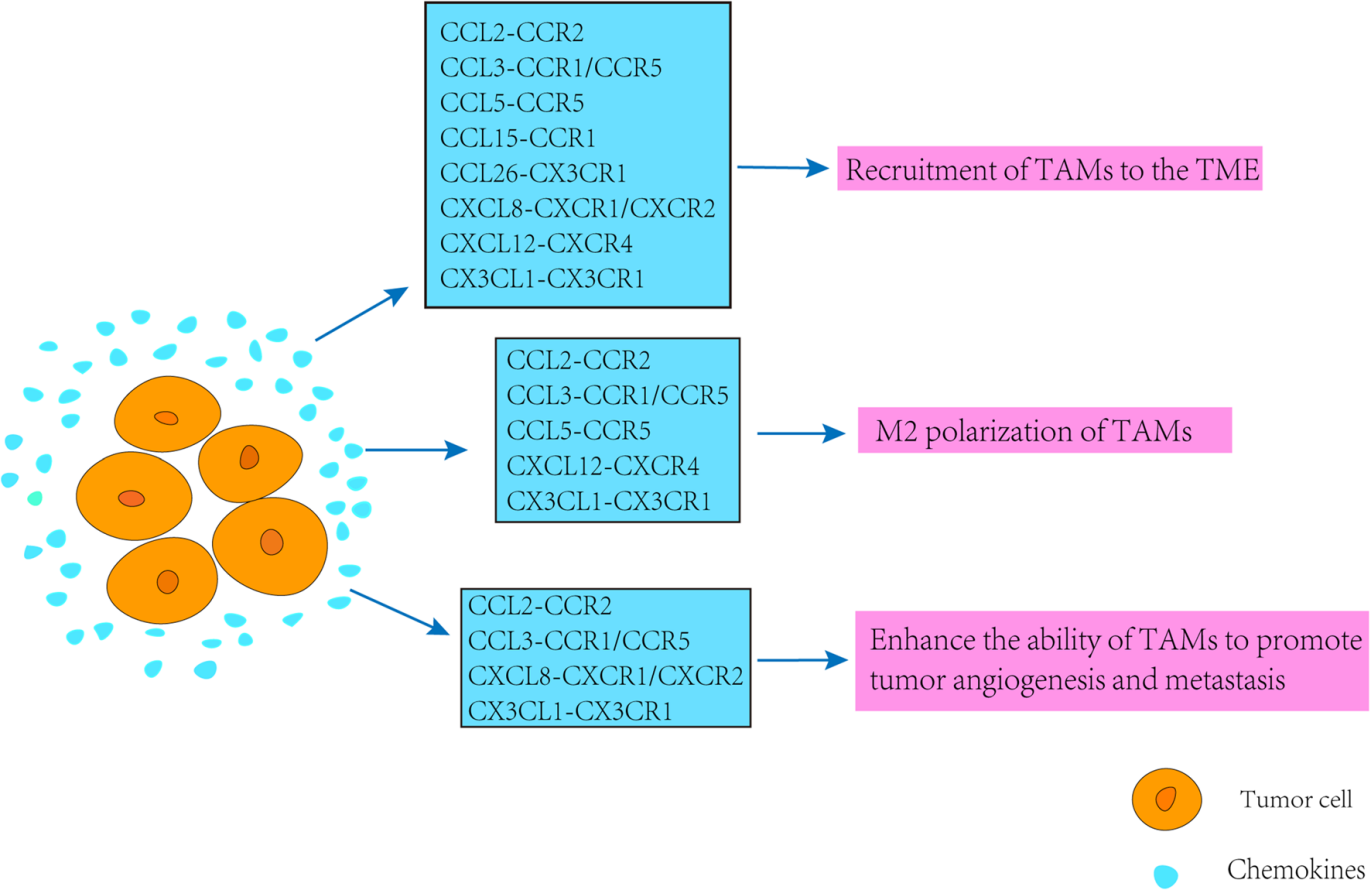
Effects of tumor-produced chemokines on TAMs

**Fig. 3:**
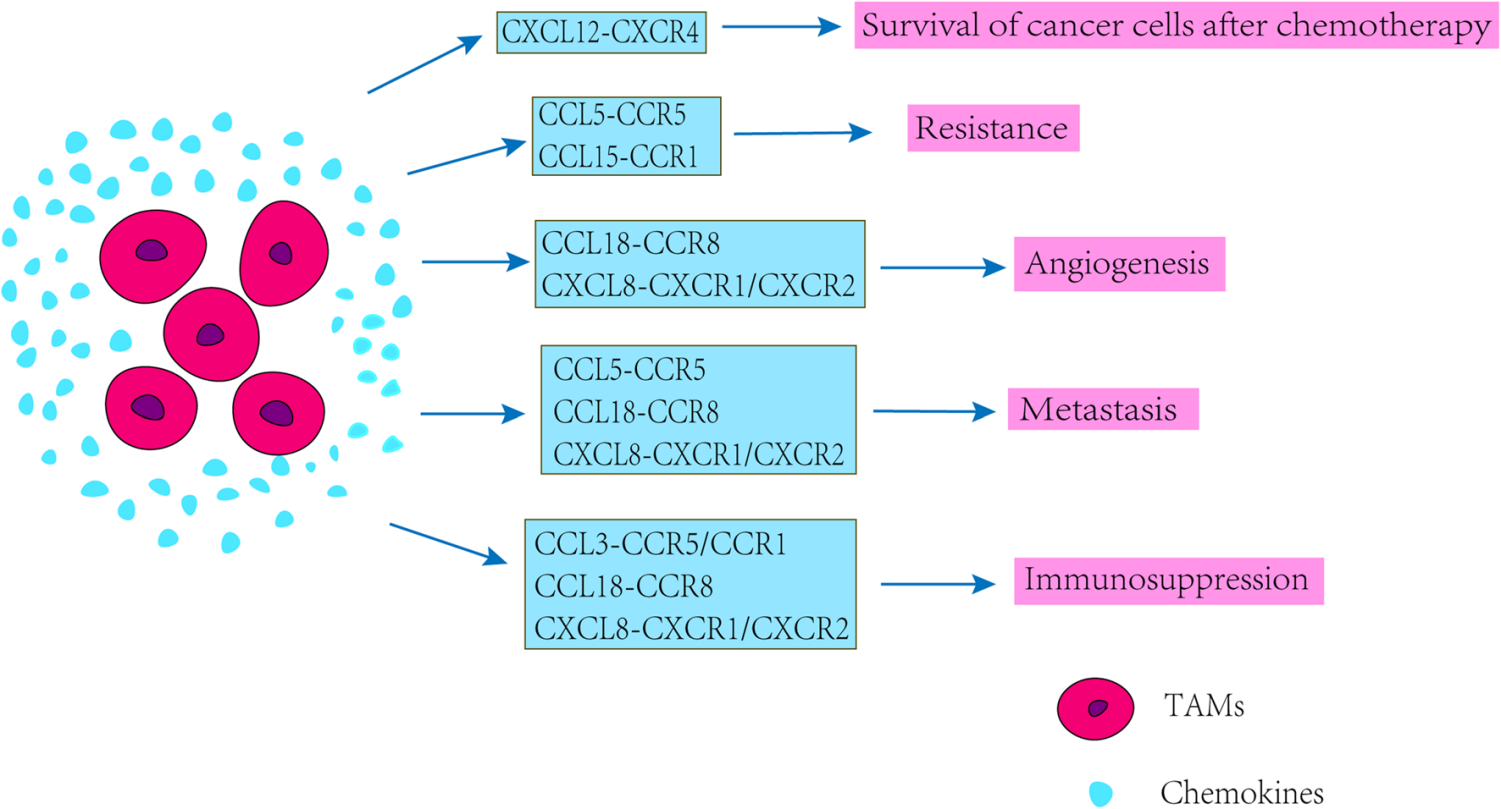
Effects of chemokines produced by TAMs on tumors

Tumor is more of a systemic disease since metastasis happens in the great majority of patients. Effectiveness achieved by existing therapeutics is far from satisfactory, since most of the current paradigms are designed to eliminate or interdict tumor cells themselves, while the successful outgrowth of metastases is largely influenced by non-malignant cells of the TME. As the major orchesters of the TME, TAMs tightly regulate tumor metastasis in all of the steps involved. In TME, TAMs, as a kind of important cells in tumor matrix, are more inclined to be M2 polarized to promote tumor development and invasion. In most cancers, including breast, lung, pancreatic, colon, kidney, head and neck, and stomach cancers, etc., M2 macrophages are the major subset of TAMs in the TME of mice and cancer patients. Chemokines and their receptors promote leukocyte migration to specific sites as well as regulate host immune responses and other physiological processes; however, this system is also thought to play a role in tumor development, progression and metastasis. Chemokines promote tumor development by regulating TAMs; meanwhile, TAMs promote tumor invasion and metastasis through chemokine-related signaling pathways (Fig. 2 and 3). Chemokines described in this review have multiple effects on TAMs in tumors, including inducing the production and infiltration of TAMs in tumors, promoting the proliferation of TAMs, promoting the tumor-promoting function of TAMs, maintaining the inhibitory function of TAMs and regulating the polarization of TAMs, etc. (Table 2). When we get a better understanding of the role of chemokines in the crosstalk of TAMs and tumor, the potential therapeutic strategies targeting chemokines would display a promising picture for cancer intervention. Indeed, we believe that targeting the pro-metastatic components of TME and reestablishing a healthier microenvironment with a reborn capacity to hamper tumor growth will certainly hold promise for cancer therapy.

**Table 2:** Effects of chemokines in the crosstalk between TAMs and tumor

Chemokines	Receptors	Cancer type	Function	Mechanism	References
CCL2	CCR2	Retinoblastoma	Recruitment of TAMs in the TME	The CCL2-CCR2 axis was activated	[26]
CCL2	CCR2	Pancreatic cancer	Formation of immunosuppressive TAMs	CCL2 recruited CCR2 + inflammatory monocytes to migrate to tumors	[27]
CCL2	CCR2	Esophageal squamous carcinoma	Blocked the recruitment of TAMs	Blocked the CCL2-CCR2 signaling pathway	[28]
CCL2	CCR2	Esophageal squamous carcinoma	Immune escape	M2 polarization increased the expression of PD-L2 in TAMs	[28]
CCL2	CCR2	Gastric cancer	Recruitment of TAMs in the TME	Increased CCL2 secretion	[29]
CCL2	CCR2	Hepatocellular carcinoma	Inflammatory monocyte recruitment and infiltration were reduced; the M2 polarization of TAMs was inhibited	CCL2 gene was knocked out or CCL2-CCR2 signaling pathway was blocked	[30]
CCL3	CCR5/CCR1	Pediatric high-grade glioma	TAMs infiltration is inhibited	Knocked out the CCL3 gene	[38]
CCL3	CCR5	Esophageal squamous cell carcinoma	Enhanced the ability of TAMs to promote the invasion and metastasis of tumor cells	The CCL3-CCR5 axis activated phosphorylation of Extracellular regulated protein kinases (ERK) and PAK	[39]
CCL3	CCR1	Gastric cancer	The recruitment of TAMs in TME increased and The M2-phenotype polarization of TAMs is promoted	CCL3-CCR1 interaction	[40]
CCL3	CCR5/CCR1	Intrahepatic cholangiocarcinoma	Adjusted the TME	M2 macrophages increased the secretion of cytokines (GM-CSF, TNF-α, Intercellular cell adhesion molecule-1 (ICAM-1), IL-6, etc.) and chemokines (CCL1, CCL3, etc.)	[41]
CCL5	CCR5	Malignant phyllodes tumor	The recruitment and repolarization of TAMs	CCL5-CCR5-driven signal cascade reaction	[47]
CCL5	CCR5	Liver cancer	M2 TAMs polarization	Through the CCL5-CCR5 signaling pathway	[48]
CCL5	CCR5	Gastric cancer	Promoted the proliferation, invasion and metastasis of tumors	TAMs secreted large amounts of CCL5	[49]
CCL5	CCR5	Prostate cancer	Promoted chemical resistance and distant metastasis of prostate cancer	TAMs-mediated STAT3-dependent epithelial–mesenchymal transformation by secreting CCL5	[50]
CCL5	CCR5	Gastric cancer	Promoted gastric cancer progression	TAMs-mediated GSN silencing by increasing the expression of DNMT1 in gastric cancer cells through the CCL5-CCR5、STAT3 signaling pathway	[51]
CCL5	CCR5	Breast cancer	Enhanced the ability of TAMs to promote tumor metastasis	Through the CCL5—CCR5 axis	[52]
CCL18	CCR8	Gallbladder carcinoma	Promoted tumor invasion and metastasis	M2 TAMs activated PI3K/Akt signaling by secreting CCL18	[57]
CCL18	CCR8	Ovarian cancer	EMT and metastasis	The M2 TAMs released CCL18	[58]
CCL18	CCR8	Colorectal cancer, Osteosarcoma, Head and neck squamous cell carcinoma, Breast cancer, Pancreatic ductal adenocarcinoma, lung cancer	Promoted tumor invasion and metastasis	M2 TAMs secreted CCL18	[59–67, 69]
CCL18	CCR8	Breast cancer	Promoted tumor angiogenesis	M2 TAMs secreted CCL18	[69]
CCL18	CCR8	Non-small cell lung cancer	Exerted immunosuppressive effect	CCL18^+^TAMs inhibited the production of inflammatory factors	[70]
CCL15	CCR1	Follicular thyroid carcinoma	Recruitment of TAMs in the TME	Tumors secreted CCL15	[77]
CCL15	CCR1	Squamous cell carcinoma of the head and neck	Developed resistance to gefitinib	Paracrined CCL15 of M2 TAMs and through the CCL15-CCR1-NF-κB pathway	[78]
CCL26	CX3CR1	Colorectal cancer	TAMs infiltration	PRL-3 raised CCL26	[83]
CXCL8	CXCR1/CXCR2	Cancer	Recruitment of TAMs in tumors	Circulating hypoxia activated HIF-1 and NF-κB in tumor cells, which led to increased production of VEGF-A, CCL2/ MCP-1, CXCL1/GRO-α, CXCL8/IL-8 and PGE2	[90]
CXCL8	CXCR1/CXCR2	Bladder cancer	Promoted tumor invasion and metastasis and immunosuppression	The invasion of TAMs in TME led to the elevation of CXCL8, which in turn promoted the secretion of MMP-9, VEGF and E-cadherin(E-Cad) by bladder cancer cells	[91]
CXCL8	CXCR1/CXCR2	Gastric carcinoma	immunosuppression	CSF-2 promoted TAMs secretion of CXCL8, which induced decreased infiltration of CD8 + T cells and increased PD-L1 expression on macrophages, thereby inhibiting CD8 + T cell activity	[92]
CXCL8	CXCR1/CXCR2	Breast cancer	Enhanced the migration, invasion and EMT ability	TAMs secreted CXCL8	[93]
CXCL8	CXCR1/CXCR2	Oral squamous cell carcinoma	Promoted the differentiation of monocyte-derived TAMs	The tumor secreted IL-8	[94]
CXCL8	CXCR1/CXCR2	Epithelial ovarian cancer	Interfered with the differentiation of monocyte-derived TAMs	Neutralizing monoclonal antibodies against IL-8 were used	[95]
CXCL12	CXCR4	Colorectal cancer	Induced TAM migration	sirtuin 1(SIRT1) passed through the CXCR4-CXCL12 pathway	[100]
CXCL12	CXCR4	Gastric carcinoma	Regulated the polarization of TAMs to M2 macrophages in tumor	pituitary transcription factor (POU Class 1 Homeobox 1, POU1F1) passed through the CXCL12-CXCR4 axis	[101]
CXCL12	CXCR4	Oral squamous cell carcinoma	Induced M2 macrophages polarization	cancer-associated fibroblasts (CAFs) passed through the CXCL12-CXCR4 signaling pathway	[102]
CXCL12	CXCR4	Ovarian cancer	Promoted M2 to M1 polarization of TAMs in tumors	The expression of CXCL12 and CXCR4 in tumor cells was downregulated	[103]
CXCL12	CXCR4	Prostate Cancer	Promoted the survival of cancer cells after chemotherapy	Increased secretion of CXCL12 by TAMs led to activation of its receptor CXCR4	[104]
CXCL12	CXCR4	Adenocarcinoma of the colon and stomach	Promoted TAMs-mediated CD8 + T cell inhibition	The activation of CXCL12-CXCR4	[105]
CXCL12	CXCR4	Cancer	Tumor cell infiltration	The unidirectional transition from migrating macrophages to perivascular macrophages is regulated by CXCL12 and CXCR4	[106]
CX3CL1	CX3CR1	Skin cancer, liver cancer	M2 TAMs were recruited	Through the CX3CL1-CX3CR1 axis	[21, 115]
CX3CL1	CX3CR1	Breast cancer, testicular reproductive carcinoma	The invasion of TAMs in the tumor	Increased CX3CL1 expression	[113, 114]
CX3CL1	CX3CR1	Cancer	TAMs infiltration was increased in TME and promoted angiogenesis	Activation of CX3CR1 inhibited TAMs apoptosis	[118]

Tumor-derived chemokines including CCL2, CCL3, CCL5, CCL15, CCL26, CXCL8, CXCL12 and CX3CL1 regulate the role of TAMs in tumors through CCR2, CCR1/5, CCR5, CCR1, CX3CR1, CXCR1/CXCR2, CXCR4 and CX3CR1, respectively, including: recruiting TAMs into the TME, modulating the M2 polarization of TAMs, enhancing the ability of TAMs to promote tumor angiogenesis and metastasis.

Chemokines secreted by TAMs, including CCL3, CCL5, CCL15, CCL18, CXCL8 and CXCL12, promote tumor metastasis, angiogenesis, cancer cell survival, immunosuppression and resistance of cancer cells after chemotherapy via CCR1/5, CCR5, CCR1, CCR8, CXCR1/CXCR2, CXCR4, respectively.

Blocking these chemokines signaling pathways can inhibit the tumor-promoting effect of TAMs, which is currently one of the research directions for preventing tumor development. Many studies have proved that many chemokine antagonists can inhibit the growth and metastasis of tumors by modulating the effects of TAMs, but there are still too few related studies and applications in clinical trials, which need to be further studied and solved. Moreover, molecular and cell biological details involved in the regulation of the action of TAMs might be more complicated than what we expect. There may be other yet-to-be-identified or well-studied chemokine receptor pairs and potential signaling pathways involved in TAMs' tumor-promoting process. There may be other mechanisms regulating the expression of chemokines and their receptors. Various major points of regulation networks remain elusive. This is crucial for the tumor-promoting effect of TAMs, and further research is needed.

The TME is a complex system composed of a plethora of cells other than TAMs, such as endothelial cells, cancer-associated fibroblasts, neutrophils, mesenchymal stem cells, MDSCs and mast cells. They and their base material around are intimately linked and interconnected with each other constantly alongside tumor progression and the formation of metastasis. Chemokines all play some role in the crosstalk between these cells and the tumor. Hence, excavating the respective roles of those parts of TME and modeling their complicated interreaction evolving along with the metastasis by system biology approaches might be the ways for future research. Meanwhile, although combining targeted agents of chemokines with other treatments is an effective approach, but many patients are less physically able to accept the simultaneous effects of multiple therapies. Combination therapies assessing patient tolerability at the time of clinical trials are highly warranted.

Some chemokines described in this review, including CCL2, CCL5 and CCL15, can also recruit effector cells (including NK cells, T lymphocytes) that are critical for tumor clearance. Targeting these chemokines may reduce the number of TAMs as well as effector cells. Therefore, weighing the pros and cons of targeting these chemokines in anticancer therapy is critical for optimal patient outcomes.

## Data Availability

Not applicable.

## Abbreviations

TME: Tumor microenvironment
TAMs: Tumor-associated macrophages
MDSCs: Myeloid-derived suppressor cells
TAN: Tumor-associated neutrophils
Tregs: Regulatory T cells
NK cells: Natural killer cells
LPS: Lipopolysaccharide
TNF-α: Tumor necrosis factor-α
TGF-β: Transforming growth factor-β
Th1: Type 1 T helper
VEGF: Vascular endothelial growth factor
GM-CSF: Granulocyte–macrophage colony-stimulating factor
TEMs: Tie2-expressing monocyte/macrophages
MMP-9: Matrix metalloprotein-9
COX-2: Cyclooxygenase-2
DCs: Dendritic cells
IM: Inflammatory monocytes
ESCC: Esophageal squamous cell carcinoma
PD-1: Programmed cell death protein 1
pHHGs: Pediatric high-grade gliomas
EMT: Epithelial–mesenchymal transition
LKN-1: Leukotactin-1
PRL-3: Regenerated liver phosphatase 3
HIF-1: Hypoxia-inducible factor 1
PGE2: Prostaglandin E2
INF-γ: Interferon-γ
SDF-1: Stromal-derived factor-1
EGCG: Epigallocatechin-3-gallate
OLA-PEG, NOX-A12: Olaptesed pegol
PF-4: Platelet-derived factor 4
MCF-7: Michigan cancer foundation-7
PDAC: Pancreatic ductal adenocarcinoma
RT: Radiotherapy
ERK: Extracellular-regulated protein kinases
ICAM-1: Intercellular cell adhesion molecule-1
E-Cad: E-cadherin
SIRT1: Sirtuin 1
POU1F1: Pituitary transcription factor
CAFs: Cancer-associated fibroblasts
